# PEG2-Induced Pyroptosis Regulates the Expression of HMGB1 and Promotes hEM15A Migration in Endometriosis

**DOI:** 10.3390/ijms231911707

**Published:** 2022-10-03

**Authors:** Yi Huang, Ruiyun Li, Rui Hu, Jia Yao, Yuan Yang

**Affiliations:** 1 The First Clinical Medical College, Lanzhou University, Lanzhou 730000, China; 2 Gansu Province Key Laboratory Biotherapy and Regenerative Medicine, Lanzhou 730000, China; 3 Reproductive Medicine Center, The First Hospital of Lanzhou University, Lanzhou 730000, China

**Keywords:** endometriosis, pyroptosis, PGE2, cell migration

## Abstract

Endometriosis (EMS) is a common gynecological disease. Prostaglandin E2 (PGE2), which induces chronic pelvic inflammation and cell pyroptosis, a form of programmed cell death based on inflammasome activation, are involved in EMS, but the extent of their involvement and roles remain unclear. The present study aimed to evaluate PGE2-induced pyroptosis in EMS and the influence of PGE2 in EMS progression. Using western blotting, it was found that the expressions of PGE2 and pyroptosis-related proteins (NLRP3, cleaved caspase-1, interleukin (IL)-1β and IL-18) were higher in EMS tissues than in normal endometrial tissues. The levels of PGE2, IL-1β, and IL-18 in the serum of patients with EMS and cell culture fluids were also detected. Using the transwell assay, we verified that PGE2 promoted hEM15A migration via the NLRP3/caspase-1 pyroptotic pathway, and PGE2-induced pyroptosis upregulated the expressions of high mobility group box 1 (HMGB1), E-cadherin, and vimentin. Immunohistochemistry analysis confirmed that PGE2-induced pyroptosis contributed to EMS invasion. These results suggest that PGE2-induced pyroptosis affects the progression of EMS by changing the migration ability of pyroptotic cells and upregulating the expression of HMGB1, E-cadherin, and vimentin. Our findings provide crucial evidence for new treatment pathways and use of anti-inflammatory drugs in EMS.

## 1. Introduction

Endometriosis (EMS) is a common gynecological disease that occurs in women of childbearing age, with an incidence of 10–15%. In patients with infertility, the incidence is approximately 40–50% [1,2]. The main clinical manifestations are dysmenorrhea, chronic pelvic pain, sexual pain, pelvic mass, and infertility. EMS presents malignant tumor characteristics, such as implantation, invasion, distant metastasis, and recurrence, and, hence, seriously affects the physical and mental health of women. Ectopic lesions themselves can lead to the occurrence of pelvic inflammation, which promotes further growth of ectopic endometrial lesions. These repeated inflammatory reactions induce an abnormal increase in inflammatory cytokines mediating the adhesion, proliferation, differentiation, and invasion of EMS lesions [3,4]. In addition, the malignant transformation of endometriosis is closely related to ovarian clear cell carcinoma and ovarian endometrioid carcinoma, and is even considered as the precancerous lesion of the latter [5,6,7]. Previous clinical studies have shown that patients with endometriosis associated ovarian carcinoma (EAOC) have some special clinicopathological features and a relatively good prognosis [8]. Basic research has found that specific genes are associated with abnormal expression [9]. However, studies on the role of endometriosis in the pathogenesis of ovarian cancer are still lacking.

Prostaglandin E2 (PGE2) is a common inflammatory factor that significantly increases in the serum of patients with EMS; therefore, it has been thoroughly studied in the pathogenesis of EMS [10]. In patients with EMS, PGE2 not only inhibits apoptosis of endometrial fragments, but also promotes cell proliferation in these fragments to form EMS lesions. In addition, elevated levels of PGE2 allow pain-causing substances and inflammatory mediators to leak out of blood vessels into local areas, causing dysmenorrhea [11]. Despite its recognized involvement in the progress of EMS, the inability of PGE2 inhibition as well as other targeted drugs in the treatment of EMS remains elusive. Moreover, previous studies have suggested that PGE2 may also be involved in other mechanisms for EMS progression.

Pyroptosis is a form of programmed cell death, based on activated inflammasome. In the cytoplasm, multiprotein complexes are formed to activate inflammatory NLR family pyrin domain-containing 3 (NLRP3), caspase-1, and caspase-4/5/11, which further lyse the perforated cell membrane of gasdermin D protein to promote pyroptosis [12,13,14]. Pyroptotic cells secrete a large number of inflammatory factors, such as interleukin (IL)-1β and IL-18. These inflammatory factors recruit inflammatory cells, induce the synthesis and release of IL-1α, IL-6, tumor necrosis factor (TNF)-α, and other substances, inducing an inflammatory response leading to various pathological processes, such as inflammatory exclusion, tissue destruction, and edema formation. It has been found that the pyroptosis-related protein NLRP3 is highly expressed at the early stage of breast cancer, increasing immune protection and inhibiting tumor progression [15]. However, when the tumor metastasizes, NLRP3 expression is significantly reduced and promotes tumor development. NLRP3 is also involved in innate immune response during cervical cancer. Reactive oxygen species activate the NLRP3 inflammasome to induce pyroptosis in cervical cancer cells, and then participate in tumor progression. Tripartite motif-containing 24 (TRIM24) may promote NLRP3/caspase-1/IL-1β-mediated pyroptosis during EMS through NLRP3 ubiquitination, revealing an important molecular mechanism [16]. Despite the crucial role of pyroptosis in EMS, no study has focused on the relationship between pyroptosis and inflammatory factors during EMS, or on whether the release of such factors triggers a series of secondary inflammatory reactions that participate in EMS progression.

The aim of the present study was to evaluate whether PGE2-induced pyroptosis is involved in EMS progression. Specifically, we examined the expression of PGE2 and pyroptosis-related proteins (NLRP3, cleaved caspase-1, IL-1β, and IL-18) in ovarian endometriotic cyst tissues. We further determined if PGE2-induced pyroptosis regulated the expression of HMGB1 and changed hEM15A migration both in vitro and in vivo. Cell migration signature proteins, E-cadherin and vimentin, were also detected. We hypothesized that PGE2 release due to pyroptosis was involved in the progression of EMS lesions by changing cell migration. The results of the present study advocate exploring new insights into the pathogenesis of EMS via inflammation and provide new treatment routes of EMS in clinical practice.

## 2. Results

### 2.1. PGE2 and Pyroptosis-Related Proteins Highly Expressed in Ectopic Ovarian Endometrium

**Figure 1:**
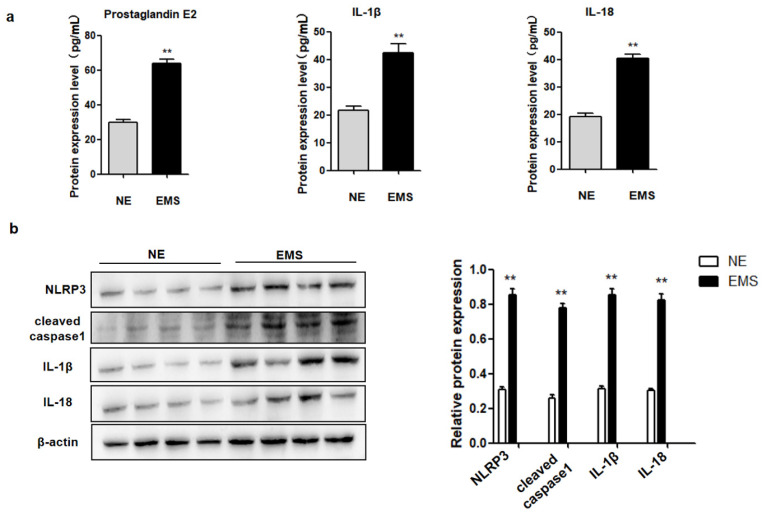
The expression of PGE2 and pyroptosis related proteins (NLRP3, cleaved caspase-1, IL-1β and IL-18) were increased in ectopic ovarian endometriosis cyst patients. (**a**) The expression of PEG2, IL-1β and IL-18 in serum of ectopic ovarian endometriosis cyst (EMS) and in the normal endometrium (NE) were detected by ELISA; (**b**) Pyroptosis related proteins (NLRP3, cleaved caspase-1, IL-1β and IL-18) protein expression were measured by Western blot analysis in NE and EMS. The data are expressed as means ± SEM. ** *p* < 0.05 by two-tailed Student’s *t* test.

Inflammatory cytokine PGE2 production and pyroptosis are common phenomena in inflammatory diseases, but there is no study on their co-expression during EMS. Therefore, the expression of PGE2 and pyroptosis-related proteins (NLRP3, cleaved caspase-1, IL-1β, and IL-18) in ectopic ovarian endometriotic cyst tissues (n = 28) and normal endometrial tissues (n = 15) were investigated. Pyroptosis-related protein levels (NLRP3, cleaved caspase-1, IL-1β, and IL-18) were increased in ectopic ovarian endometriotic cyst tissues, compared to those in normal endometrial tissues (Figure 1a). Furthermore, PGE2, IL-1β, and IL-18 levels were elevated in the serum of patients with EMS, compared to those in normal controls (Figure 1b). These data suggested PGE2 may be related to pyroptosis during the occurrence and development of ovarian endometriotic cysts.

### 2.2. PGE2 Induces hEM15A Pyroptosis

**Figure 2:**
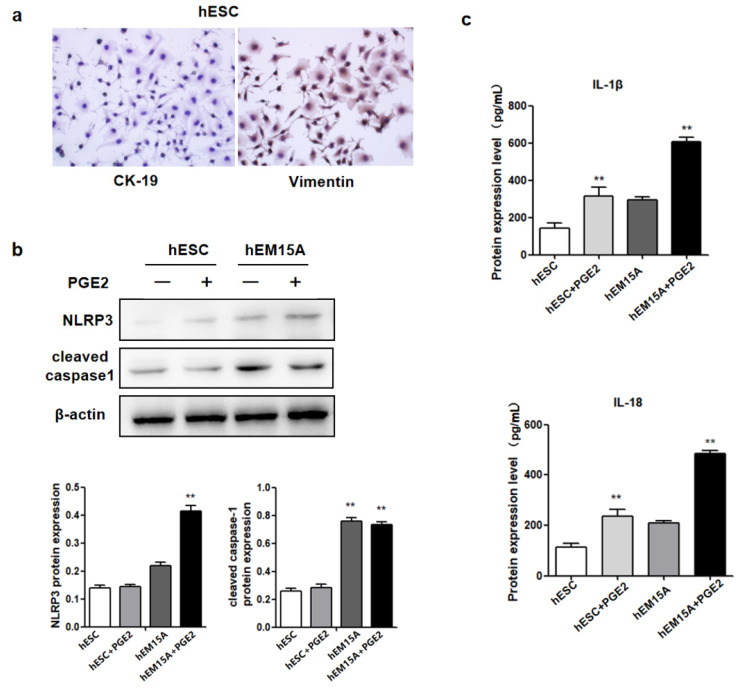
PGE2 induced hEM15A pyroptosis. (**a**) After added 75 ng/mL PGE2 for 24 h in hEM15A and hESC, the protein expression of NLRP3, cleaved caspase-1, IL-1β and IL-18 were detected by Western blot analysis. (**b**) The level of IL-1β and IL-18 in cell culture medium were detected by ELISA in hEM15A and hESC. The data are expressed as means ± SEM. ** *p* < 0.05 by one-way ANOVA. (**c**) ELISA was used to detect the expression of IL-1β and IL-18 in hESC and hEM15A without and after PGE2 treatment.

To determine whether PGE2-induced NLRP3/caspase-1/IL-1β-mediated pyroptosis is involved in EMS, hEM15A and hESCs were treated with 75 ng/mL PGE2. After 24 h, the expression of NLRP3 and cleaved caspase-1 was upregulated in hEM15A compared to that in hESCs (Figure 2b). The levels of IL-1β and IL-18 in the cell culture medium were significantly increased in hEM15A compared to those in hESCs (Figure 2c). These results indicated that PGE2 induced pyroptosis in hEM15A cells but not in hESCs.

### 2.3. PEG2 Promotes hEM15A Migration

**Figure 3:**
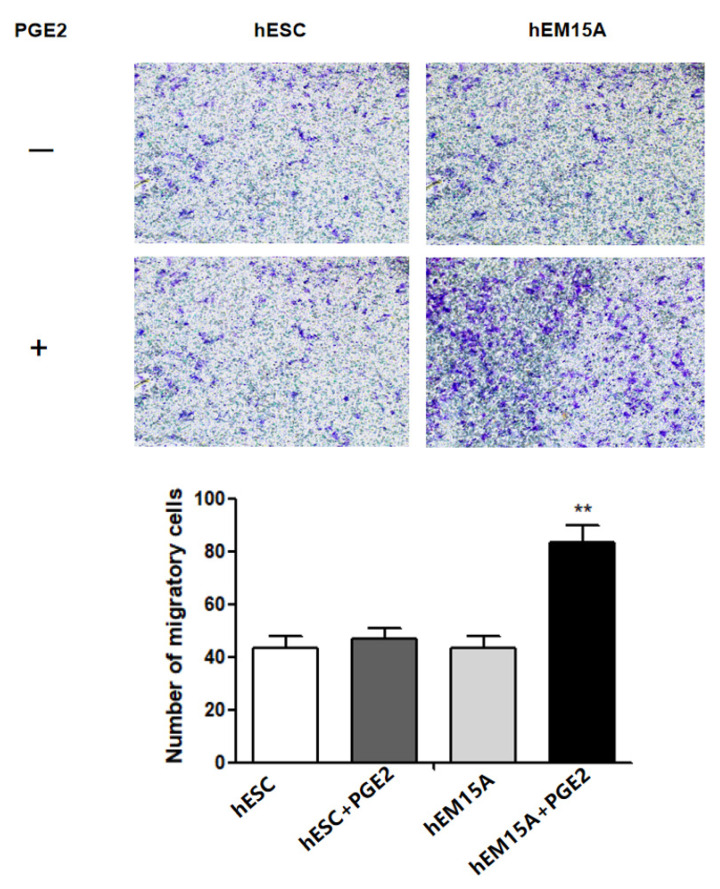
After added 75 ng/mL PGE2 for 24 h in hEM15A and hESC, Transwell migration assay was to evaluate the effect of PGE2 on the migration of hESC and hEM15A.The data are expressed as means ± SEM. ** *p* < 0.05 by one-way ANOVA.

It has been shown that EMS has similar properties to malignant tumors with enhanced cell migration and invasion. Therefore, we further evaluated the effect of PGE2 on hESCs and hEM15A migration using the transwell assay. After 24 h, the 75 ng/mL PGE2-treated hEM15A cells showed significantly higher cell migration than that by hESCs, suggesting that PGE2 is involved in hEM15A migration (Figure 3).

### 2.4. PEG2 Promotes hEM15A Migration through NLRP3/Caspase-1 Pyroptotic Pathways

**Figure 4:**
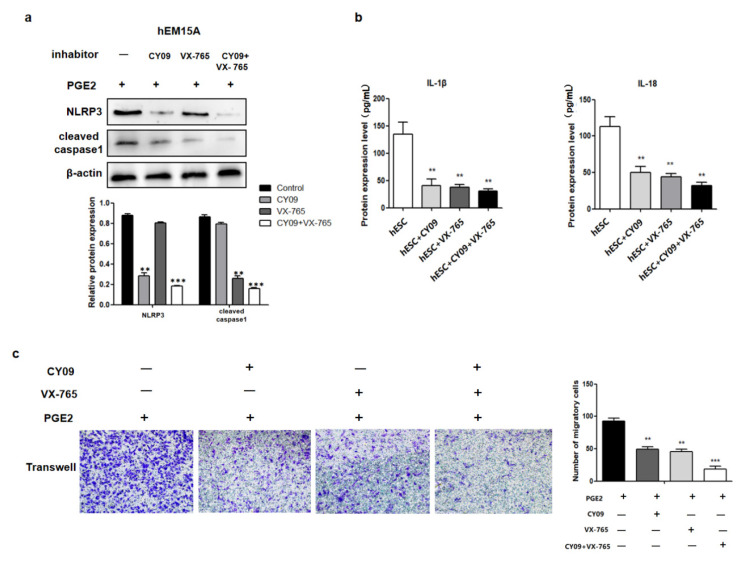
PEG2 promoted hEM15A migration through NLRP3/caspase-1 pyroptosis pathways. (**a**) In hEM15A, PGE2, co-cultured PGE2 + CY-09 (the inhibitor of NLRP3), co-cultured PGE2 + VX-765 (the inhibitor of caspase-1) and co-cultured PGE2 + CY-09 + VX-765, pyroptosis related proteins (NLRP3 and cleaved caspase-1) were detected by Western blot analysis. (**b**) In hEM15A, PGE2, co-cultured PGE2 + CY-09 (the inhibitor of NLRP3), co-cultured PGE2 + VX-765 (the inhibitor of caspase-1) and co-cultured PGE2 + CY-09+VX-765, the level of IL-1β and IL-18 in cell culture medium were detected by ELISA. (**c**) In hEM15A, PGE2, co-cultured PGE2 + CY-09 (the inhibitor of NLRP3), co-cultured PGE2 + VX-765 (the inhibitor of caspase-1) and co-cultured PGE2 + CY-09 + VX-765 were evaluated by Transwell migration assay in different groups.** *p* < 0.05, *** *p* < 0.001 by one-way ANOVA.

The relationship between cell migration and pyroptosis were then evaluated in hEM15A cells co-cultured in PGE2 and CY09 (the inhibitor of NLRP3). Pyroptosis-related proteins (NLRP3 and cleaved caspase-1) and the levels of IL-1β and IL-18 in the cell culture medium were decreased compared to that in PGE2-treated cells alone (Figure 4a,b). In addition, the migration ability of hEM15A cells under the effect of PGE2 and CY09 was significantly lower than that of PGE2-treated cells alone (Figure 4c). Similarly, in co-cultured PGE2 and VX-765 (the inhibitor of caspase-1) cells, cleaved caspase-1 and the levels of IL-1β and IL-18 in cell culture medium were decreased compared to that of PGE2-treated cells alone (Figure 4a,b). The migration ability of hEM15A cells under the effect of PGE2 and VX-765 was also significantly lower than that of PGE2-treated cells alone (Figure 4c). When both CY09 and VX-765 were added, PGE2 did not activate the expression of pyroptosis-related proteins (NLRP3, cleaved caspase-1, IL-1β, and IL-18), and the migration ability of hEM15A cells was only slightly activated. Thus, CY09 or/and VX-765 reduced the migration ability of hEM15A cells after PGE2 induction, and pyroptosis was involved in the process, in that PGE2 activated EMS invasion.

### 2.5. PGE2-Induced Pyroptosis Regulates the Expression of High Mobility Group Box 1 (HMGB1), E-Cadherin, and Vimentin

**Figure 5:**
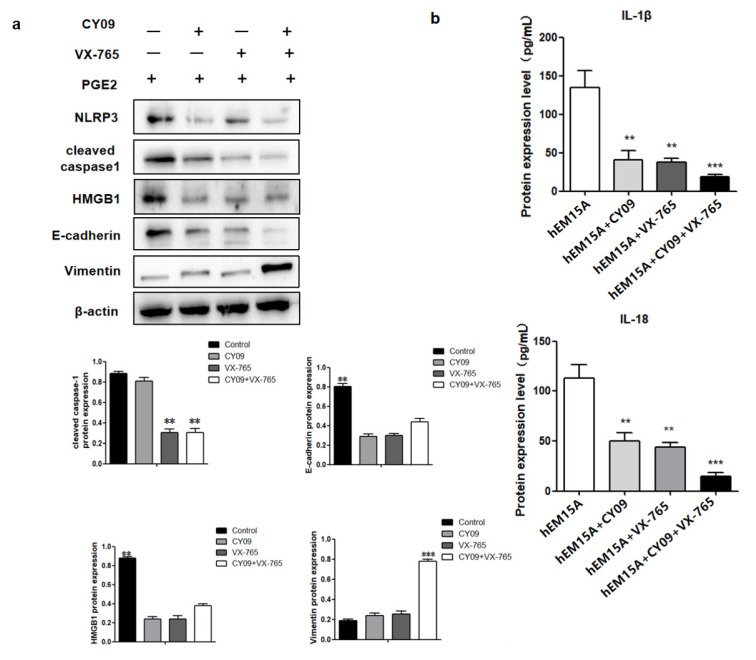
PEG2-induced pyroptosis regulates the expression of HMGB1 and promotes hEM15A migration ability. (**a**) In hEM15A, PGE2, co-cultured PGE2 + CY-09 (the inhibitor of NLRP3), co-cultured PGE2 + VX-765 (the inhibitor of caspase-1) and co-cultured PGE2 + CY-09 + VX-765, the protein of NLRP3, cleaved caspased1, HMGB1, E-cadherin and Vimentin were detected by Western blot analysis. (**b**) In hEM15A, PGE2, co-cultured PGE2 + CY-09 (the inhibitor of NLRP3), co-cultured PGE2 + VX-765 (the inhibitor of caspase-1) and co-cultured PGE2 + CY-09 + VX-765, the level of IL-1β and IL-18 in cell culture medium were detected by ELISA in hEM15A and hESC. The data are expressed as means ± SEM. ** *p* < 0.05, *** *p* < 0.001 by one-way ANOVA.

In hEM15A cells co-cultured in PGE2 and CY09, HMGB1 and E-cadherin proteins had low expresssion while vimentin was highly expressed when compared to that in PGE2-treated cells alone. Similarly, co-cultured PGE2 and VX-765 cells showed low expressions of HMGB1 and E-cadherin proteins and high expression of vimentin when compared to PGE2-treated cells alone. When both CY09 and VX-765 were added, PGE2 did not influence the expressions of HMGB1, E-cadherin, and vimentin in hEM15A cells (Figure 5b).

### 2.6. PGE2-Induced Pyroptotic Pathways and Invasion of EMS Lesions In Vivo

**Figure 6:**
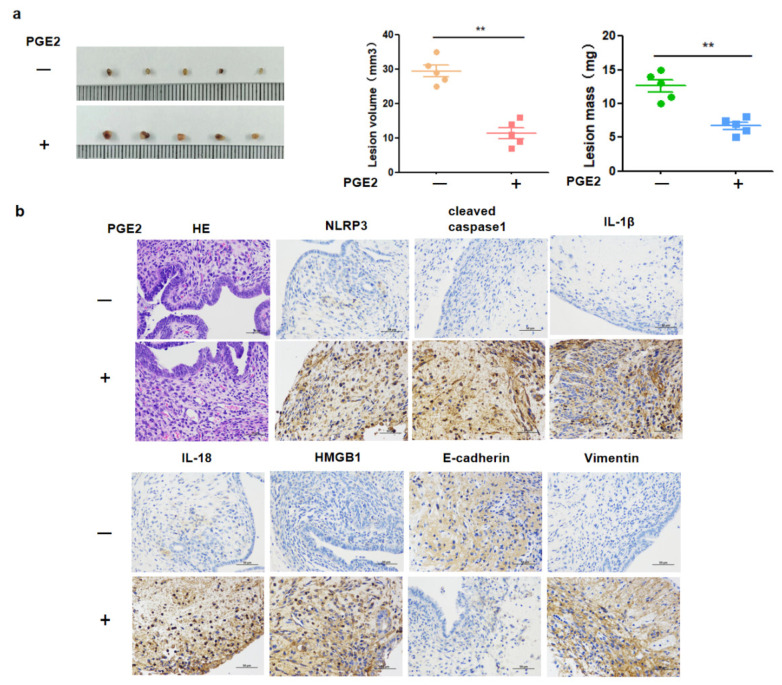
PEG2-induced pyroptosis pathways and invasion of EMS lesion in vivo. (**a**) After intervention with PGE2 in endometriosis mouse model, ectopic lesions were enlarged. Ectopic lesions obtained from endometriosis mice after continuous treatment with PGE2. The volume of ectopic lesions was statistically analyzed (** *p* < 0.05); (**b**) immunohistochemistry showed that NLRP3, cleaved caspase-1, Il-1β, Il-18, HMGB1 and Vimentin were highly expressed in the endometrial tissues of mice treated with PGE2.E-cadherin was down-regulated in the endometrial tissues of mice treated with PGE2 (** *p* < 0.05).

To further verify the induced pyroptosis effects of PGE2 on EMS in vivo, an EMS mouse model was built. As expected, PGE2 significantly induced the pyroptosis of EMS lesions. The weight and diameter (size) of EMS lesions in the PGE2 group were larger than that in the control group (Figure 6a). Additionally, pyroptosis-related proteins (NLRP3, cleaved caspase-1, IL-1β, and IL-18), HMGB1, E-cadherin, and vimentin were tested by immunohistochemistry analysis. The expression of NLRP3, cleaved caspase-1, IL-18, IL-1β, HMGB1, and vimentin was increased with increasing concentrations of PGE2 (Figure 6b). Moreover, the expression of HMGB1, E-cadherin, and vimentin was positively correlated with the expression of pyroptosis-related proteins, which was consistent with the results obtained in the in vitro experiments.

## 3. Discussion

EMS is a progressive inflammatory disease that affects women’s health and has become a major threat to fertility and individual quality of life. Two important factors of EMS to human health are ectopic lesion-induced inflammation and lesion invasion/metastasis, which are not only a key factor in disease progression, but also a root cause of recurrence after surgical and medication treatments of EMS. Previous research has shown that persistent inflammation promotes local tissue fibrosis and disease progression [17]. Therefore, exploring the relationship between inflammation and disease aggressiveness is critical to understand the underlying molecular pathogenesis of EMS and to find novel therapeutic targets. The present study described the mechanism by which PEG2-induced pyroptosis regulates the migration of EMS cells (Figure 6). A notable increase in PGE2 and pyroptosis-related proteins (NLRP3, pro-caspase-1, caspase-1, IL-1β, and IL-18) was found in EMS tissue compared with that in normal tissue, and we demonstrated that PGE2 can induce pyroptosis in EMS cells, while PGE2-induced pyroptosis can affect cell migration and upregulate the expression of HMGB1 and vimentin. These results indicated that PEG2 could significantly promote EMS invasion, activated by PEG2, which was accomplished via pyroptosis induction.

PGE2 can activate multiple anti-apoptotic factors, such as Bcl-2, and prolong cell life and accelerate ectopic lesions growth. PGE2 stimulates protein kinase A and promotes the phosphorylation of Ras-like estrogen-regulated growth to induce the proliferation of ectopic lesions. In addition, PGE2 leads to the formation of large neovascularization in ectopic lesions and provides rich blood and nutrients for ectopic lesions, which is conducive to endometrial implantation and lesion growth. The present study is, to the best of our knowledge, the first to clarify the inducing effect of PGE2 on pyroptosis, promoting cell migration and the progress of EMS. It is, therefore, of great significance for further understanding PGE2 pathogenesis in EMS.

Inflammatory PGE2 induces the expression of large amounts of IL-1β and IL-18 inflammatory factors through the pyroptotic pathway, which is a positive cycle of inflammatory response in the EMS, leading to subsequent inflammation. Inflammation is a complex and huge network. In tumors, inflammatory cells widely receive proliferative signals by releasing inflammatory factors, leading to rapid cell proliferation and differentiation and increasing the risk of cell cancerization [18,19,20]. Additionally, inflammatory cells release chemokines and cytokines, which affect the cancerous organ and regulate the growth, migration, and differentiation of cells in the tumor microenvironment [21,22,23]. In the tumorigenesis process, tumor cells promote tumor spread and metastasis through inflammation by releasing TNF-𝛼, IL-6, IL-1, and interferon factors, which in turn stimulate tumor cell growth, motility, and invasion [24,25].

In EMS, periodic bleeding occurs in the ectopic endometrium along with the menstrual cycle, which forms a wound with repeated injury and continuous healing. In the repair process, multiple systems, such as the inflammatory system, coagulation system, and immune system are often involved. The inflammatory response leads to the production of pro-inflammatory factors, and this inflammatory microenvironment promotes the proliferation, migration, metastasis, and angiogenesis of lesions through a series of pathways. It has been hypothesized that in PGE2-induced pyroptosis cells secrete a large number of IL-1β and IL-18 inflammatory factors. On the one hand, these inflammatory factors recruit more inflammatory cells, induce the synthesis and release of other factors such as IL-1α, IL-6, and TNF-α expanding the inflammatory response, and are widely involved in various pathological injury processes such as inflammatory exclusion, tissue destruction, and edema formation. In pneumonia, for instance, IL-1β and IL-18 can activate inflammatory cells, such as macrophages to release inflammatory mediators, causing inflammatory responses characterized by infiltration of macrophages and granulocytes in the lung [26]. On the other hand, inflammatory factors promote the proliferation and migration of cells. For example, IL-1β promotes the proliferation, migration, angiogenesis, and release of HMGB1 in smooth muscle cells, while IL-18 promotes the migration of breast cancer cells by down-regulating claudin-12 and inducing the P38/MAPK pathway [27]. In the present study, cleaved caspase 1, IL-1β, and IL-18 were activated after PGE2 stimulation. After PGE2-induced pyroptosis, hEM15A cell migration was increased, HMGB1 and vimentin expressions were upregulated, and E-cadherin expression was downregulated. Thus, in EMS, PGE2-induced pyroptosis seems to promote the expression of inflammatory factors that alter cell migration accompanied by abnormal expression of E-cadherin and vimentin (the markers of cell migration). To the best of our knowledge, this phenomenon was observed in EMS in this study for the first time. Although its specific regulatory mechanism needs to be further studied, the present findings contribute to revealing the underlying molecular pathogenesis of EMS and to the search for new therapeutic targets.

In general, inflammation is a double-edged sword. While protecting the body against disease, it also contributes to the progress of the disease. In the present study, the effect of PGE2-induced pyroptosis on the progression of EMS was mainly reflected in the changed migration ability of pyroptosis cells and upregulated expressions of HMGB1 and vimentin, as verified in in vivo experiments. The potential relationship found between cell pyroptosis and migration provides crucial evidence for new routes in the clinical development and use of anti-inflammatory drugs.

## 4. Materials and Methods

### 4.1. Patients and Tissues

This study was approved by the Ethics Committee of the First Hospital of Lanzhou University (Lanzhou, China), and informed consent was obtained from each patient. Ectopic ovarian endometriotic cyst tissues were obtained from patients with EMS, who underwent laparoscopic treatment in Lanzhou University First Hospital from January 2020 to January 2021 (n = 28). Normal endometrial tissue was collected from women of childbearing age observed in clinical practice (n = 15). Collected tissues were rapidly placed in liquid nitrogen and then stored at −80 °C until analyses. The following were inclusion criteria: (1) patients diagnosed as EMs by laparoscopic surgery; (2) normal hormone level and regular menstrual cycle’ (3) no history of hereditary or familial diseases, normal chromosomes, and infectious disease tests were negative. The following were exclusion criteria (1) receipt of hormone or immunosuppressive therapy in the last 3 months; (2) other endocrine diseases; (3) recurrent EMS; (4) other gynecological diseases, abnormal uterus, polycystic ovary syndrome and low ovarian response or malignant tumors. Control group: Fifteen patients with malformed mediastinal uterus who underwent hysteroscopic surgery during the same period were selected, and their normal endometrium was taken as the control group.

### 4.2. Cell Culture

The hEM15A cell line was purchased from China Center for Typical Cultures Preservation (Wuhan, China) and was cultured at 5% CO_2_ and 37 °C in RPMI/1640 (HyClone, Logan, UT, USA) containing 15% fetal bovine serum (FBS) (Gibco, Thermo Fisher Scientific, Waltham, MA, USA). Primary human endometrial stromal cells (hESCs) were isolated and cultured as follows: the endometrial tissue was washed with phosphate-buffered saline (PBS) three times within 30 min to remove blood stains. Then, the tissue was cut into pieces using a sterile ophthalmic scissor, and 3–5 mL of 0.1% type I collagenase digestion solution (Solarbio, Beijing, China) was added to the tissue fragments. After incubation in a water bath at 37 °C for 60 min, Dulbecco’s modified Eagle’s medium/nutrient mixture F-12 (DMEM/F12) medium (HyClone) containing 10% FBS and 1% penicillomycin (Solarbio) was added. The tissue suspension was then filtered through a 40-mesh screen to discard the residue of tissue retained on the mesh. After centrifugation for 10 min at 1500 rpm, the obtained cell precipitates were suspended in the complete culture medium, inoculated in Petri dishes, and incubated at 37 °C and 5% CO_2_. Cell adherence was observed after 12 h. The original medium was replaced with a fresh medium.

### 4.3. Transwell Assay

Different cell suspensions were added into the upper chamber. RPMI/1640 or DMEM/F12 containing 20% FBS was added to the lower chamber. Then, cells were cultured at 37 °C and 5% CO_2_ for 24 h. The submembrane cells were fixed with 4% paraformaldehyde, stained with 0.1% crystal violet, and the inserts were cleaned with PBS. After drying, cells were selected and counted under a light microscope.

### 4.4. Immunocytochemistry

Cell suspensions (1 × 10^6^ cells/mL) were inoculated in a six-well plate, covered with sterile cover glass, and incubated for 24 h. When cell confluence reached 80%, cells were fixed in 4.0% paraformaldehyde and permeated using 0.1–0.2% polyethylene glycoloctylphenylether. After incubation for 15 min, 5.0% normal goat abandoned blood serum was added. Anti-CK19 (1:50; Abcam, Cambridge, UK) and anti-vimentin (1:50; Abcam) were added and a negative control (PBS instead of primary antibody) was set up simultaneously; all samples were placed in wet box at 4 °C overnight. After rewarming on the next day, diluted secondary antibody was added, incubated at 37 °C for 15 min, and dyed using the SP method (i.e., adding horseradish-labeled chain enzyme lactalbumin working solution). The neutral gum was then sealed and observed under a light microscope.Immunohistochemistry analysis showed that CK19 was negative and vimentin was positive in hESCs and hEM15A. CY09 and VX-765 were purchased from Selleck (Shanghai, China).

### 4.5. Enzyme-Linked Immunosorbent Assay (ELISA)

The concentrations of PGE2, IL-1β, and IL-18 in the serum of patients and cell culture medium were detected using ELISA kits (Solarbio), according to the manufacturer’s protocol. The optical density (OD) at 450 nm was used to calculate the concentrations of PGE2, IL-1β, and IL-18.

### 4.6. Western Blot

Cells were collected and lysed. Total protein was extracted and quantified using the BCA Protein Assay Kit (Solarbio), following the manufacturer’s instructions. Primary antibodies (all Abcam) used included anti-NLRP3 (1:1000), anti-cleaved caspase-1 (1:1000), anti-IL-1β (1:1000), anti-E-cadherin (1:1000), anti-vimentin (1:1000), and anti-β-actin (1:5000) and were incubated at 4 °C overnight. After washing with tris-buffered saline with Tween (TBS-T) three times, the secondary antibody (Solarbio) was added for 1 h at room temperature. The ECL substrate (Solarbio) was used to detect the expression of target proteins in the FUSION FX5 imaging system (Bio-Rad, Hercules, CA, USA).

### 4.7. Immunohistochemistry

Paraffin-embedded tissues were sectioned, placed for 20 min in a 60 °C constant temperature box after dewaxing hydration, and subjected to high-pressure repair for 2.5 min. After washing three times with PBS (5 min each), endogenous peroxidase blocking agent was added at room temperature for 10 min. The PBS washing step (5 min each) was performed for 1 h and conducted three times. Primary antibodies (anti-NLRP3, 1:100; anti-cleaved caspase-1, 1:100; anti-IL-1β, 1:100; anti-IL-1β, 1:100; anti-E-cadherin, 1:200; anti-vimentin, 1:200; and anti-β-actin, 1:500; all Abcam) were incubated overnight. After PBS washing for 5 min three times, secondary antibodies (Solarbio) were incubated before 3,3′-diaminobenzidine (DAB) was added as the chromogen. Tissue sections were then observed and photographed under the microscope.

### 4.8. Animals

Specific pathogen free female BALB/C mice (4–5 weeks old) were purchased from the Experimental Animal Center of Lanzhou University Medical College (Lanzhou, China). Animals were raised in the laboratory animal center for 1 week before the onset of the experiments. Recipient and donor mice were subcutaneously injected with estradiol (100 μg/kg body weight) once a week for modeling until the end of the experiment. On the day of mouse modeling, both uterine horns of donor mice were extracted and placed in a glass dish containing PBS to remove fat, blood, and other tissues. The ratio of donor mice to recipient mice was 1:2. The uteri of donor mice were cut into 1–2 mm pieces and then divided into several parts of equal weight, which were injected into the abdominal cavity of recipient mice. EMS recipient mice were randomly divided into two groups (n = 5 per group), (I) control group (0.9% sodium 0.01 mL/g/day) and (II) PGE2 group (0.01 mL/g/day), which were intraperitonially injected with the respective treatment. The mice weight was measured every 3 days until day 21. After the experiment, the mice were sacrificed. The weight and size of lesions were determined.

### 4.9. Statistical Analyses

All graphics were generated using GraphPad Prism 8.0. Data are expressed as mean ± standard error of the mean (SEM) and were analyzed in SPSS 22.0 software. Two-tailed unpaired t-test was used to analyze the differences between the two groups, and one-way analysis of variance (ANOVA) was used for statistical comparisons. *p* < 0.05 was considered statistically significant.

## Data Availability

Not applicable.
